# Infectious disease surveillance system descriptors: proposal for a comprehensive set

**DOI:** 10.2807/1560-7917.ES.2020.25.27.1900708

**Published:** 2020-07-09

**Authors:** Julien Beauté, Bruno Christian Ciancio, Takis Panagiotopoulos

**Affiliations:** 1 European Centre for Disease Prevention and Control (ECDC), Stockholm, Sweden; 2 Department of Public Health Policies, School of Public Health, University of West Attica, Athens, Greece

**Keywords:** surveillance, public health surveillance, methods, standards, metadata

## Abstract

To tailor a surveillance system to its objectives and to evaluate its fitness for purpose, an accurate description of its structural elements is essential. Existing recommendations for setting up a system seldom offer a comprehensive list of all surveillance elements to be considered. Moreover, there is sometimes confusion in the way terms describing these elements are interpreted. The objective of this paper is to propose a comprehensive set of surveillance system descriptors that can delineate the important elements and clarify the meaning of the terms used. We identified 20 descriptors that we classified in five categories: (i) surveillance scheme; (ii) population and cases; (iii) supplementary data; (iv) information flow; and (v) period of time. We tried to make the definitions of these descriptors as clear and simple as possible to avoid confusion or misinterpretation of the terms used. The relative importance of each element may vary depending on the objectives of the surveillance scheme. Surveillance descriptors should be reviewed periodically to document changes and to assess if the system continues to be fit for purpose. Together with the minimum requirements for variables and the planned outputs for disseminating the data, the surveillance descriptors can be used to define surveillance standards.

## Background

Surveillance is usually defined as the systematic and continuous collection, management, analysis, interpretation and reporting of disease data to drive public health action [1,2]. Infectious disease surveillance played a major role in shaping the modern concept of surveillance, which Langmuir described as ‘the current and accurate two-way flow of information among those who need to know’ [3]. Yet, this general statement does not address a widening range of objectives [4], including descriptive disease epidemiology, outbreak detection, impact assessment of disease prevention and control interventions, research, and information for healthcare provision. Public health authorities design their surveillance system based on their objectives. A surveillance system consists of both processes, e.g. data collection, and structural elements, e.g. information technologies supporting the data collection [4]. To tailor a surveillance system to its objectives and to evaluate its fitness for purpose, an accurate description of its structural elements is essential. Inconsistent use of a surveillance system, i.e. structure and/or processes not driven by objectives, can compromise its very existence. Existing recommendations for setting up a system seldom offer a comprehensive list of all surveillance elements to be considered [5]. In addition, there is sometimes confusion in the way terms describing these elements are interpreted. For example, ‘active reporting’ and ‘active case-finding’ can be confused or misinterpreted. New data sources, e.g. electronic health records and whole genome sequencing data, pose additional challenges in the description of surveillance systems. Last, surveillance systems need to be regularly evaluated and adjusted. A thorough description of a surveillance system is often the first step of its evaluation [6,7].

The objective of this paper is to propose a comprehensive set of surveillance system descriptors that can delineate all the important elements and clarify the meaning of the terms used.

## Surveillance standards

Surveillance system descriptors should be used in defining surveillance standards for a disease or a group of diseases. Surveillance standards are a set of minimum characteristics of the system that are necessary for meeting surveillance objectives. The World Health Organization (WHO)-recommended standards for vaccine-preventable disease (VPD) surveillance include ‘the design, methods and data elements necessary for achieving the specific goals of immunization programmes’ [8]. In others terms, these standards include the surveillance system descriptors (design and methods) and the variables to be collected (data elements). To these two fundamental components, we suggest adding a third one, which would include both analyses to be performed and outputs to be produced routinely. These would cover the last essential parts of surveillance, i.e. analysis, interpretation and reporting. For example, if we consider a surveillance system for a VPD with an elimination target, surveillance standards would suggest a comprehensive system with national coverage to detect all cases. The list of variables to be collected would probably include age, vaccination and importation status. Completeness for these key variables should be high (> 90%). Reports should be frequent, possibly monthly, for basic descriptive analyses.

## Surveillance descriptors

### Revision of The European Surveillance System (TESSy) data sources

For the purpose of this paper, we focused on proposing a comprehensive set of surveillance system descriptors. We only considered indicator-based surveillance, as opposed to event-based surveillance [9], relying on healthcare-based information that depends on healthcare professional recognition of disease, diagnosis or suspicion. As a starting point, we took the data source description of The European Surveillance System (TESSy) [10]. We revisited all these descriptors, revised their definitions and complemented them when necessary. During this process we followed a few guiding principles. First, we aimed for comprehensiveness. We wanted the list of indicators to be as exhaustive as possible to capture all relevant elements of a surveillance system. We also wanted to allow flexibility since some elements may have more importance than others for a given surveillance system. Second, we strived for simplicity by excluding any redundancy or elements deemed to be of little relevance. Last, we endeavoured for clarity by selecting self-explanatory terms, respecting accepted or traditional use of terms. We identified 20 descriptors that we classified in five categories (Table): (i) surveillance scheme; (ii) population and cases; (iii) supplementary data; (iv) information flow; and (v) period of time. In the following paragraphs we review the selected descriptors and their explanation.

**Table:** Proposed surveillance system descriptors, description categories, characteristics and comments for describing infectious disease surveillance systems

Category	Number	Descriptor	Type/Characteristics	Comments
**Surveillance scheme**	1	System design	(i) Comprehensive: All healthcare providers of at least one level of care in a defined geographical area, e.g. all general practitioners of the region, should report their cases. (ii) Sentinel: Only a subset of healthcare providers should report their cases. (iii) Other: This option should be used when there is a combination of the previous categories, e.g. comprehensive in one part of the year or the country and sentinel in another.	Not to be confused with number 8 (Geographical coverage) and 9 (Population under surveillance). If the selection of sentinel providers is designed on a representative sample, it is possible to calculate rates.
	2	Mode of reporting	(i) Passive reporting: A passive surveillance system is based on data received from data providers without surveillance units prompting them to report. (ii) Passive with zero reporting: Passive reporting with the additional element of sending a report in defined time-intervals even if there are no cases to report; in this situation, zero cases are reported. (iii) Active reporting: An active surveillance system requires surveillance units to systematically take the initiative to communicate periodically with data providers to prompt them to report data. (iv) Automated data transfer: All diagnosed cases and the necessary information about them are transferred automatically from electronic healthcare records or laboratory information management systems to public health authorities for surveillance purposes. (v) Other: This includes situations in which a combination of the previous categories applies, e.g. one mode of reporting in one part of the country and another in other parts.	Active reporting should not be confused with ‘active case-finding’, which refers to the process of case identification or detection (see number 10). Active reporting refers to the reporting process.
	3	Data format	(i) Case-based: Each individual case of the disease under surveillance is reported separately. (ii) Case-based, aggregate reporting to higher levels. (iii) Aggregate: Only the total number of cases of the disease under surveillance is reported, possibly broken down by age, sex and/or other criteria.	None
	4	Legal status of reporting	(i) Mandatory: The surveillance system has a legal or regulatory basis, at the national administrative level or other, under which reporting of cases of the disease under surveillance is compulsory. (ii) Voluntary: Reporting of cases is voluntary. (iii) Other: Any system that does not fall under one of the above descriptions, e.g. part of project, special agreement, etc..	None
	5	Temporal continuity	(i) Routine-all year: The surveillance scheme is continuously in place all year round. (ii) Routine-seasonal: The surveillance scheme is continuously in place during a predefined period every year, e.g. influenza surveillance season from week 40 to week 20 of the following year. (iii) Ad hoc surveillance system: The surveillance scheme is in place provisionally, under certain circumstances. (iv) Survey: The surveillance is based on surveys carried out on a regular basis.	None
	6	Data sources	(i) All healthcare: All healthcare providers are required to report cases to the surveillance system. (ii) Laboratories: Only laboratories are required to report cases to the surveillance system (laboratory surveillance). (iii) Specific setting: Only special healthcare services, e.g. paediatric departments, are required to report cases to the surveillance system. This includes services operating in special settings such as prisons. (iv) Other: Any other type or combination of types of health services are required to report cases to the surveillance system.	None
	7	Type of information reported	(i) Clinical, epidemiological and laboratory information is included in the data to be reported. (ii) Clinical and epidemiological information is included in the data to be reported. (iii) Laboratory and epidemiological information is included in the data to be reported. (iv) Only laboratory information is included in the data to be reported. (v) Other: Combination of types of information other than the above.	Clinical information: information related to the clinical manifestations of a disease. Epidemiological information: information related to the epidemiological context, including a link to other cases, a source of infection or an outbreak situation. Laboratory information: information related to laboratory findings, both diagnostic methods and results.
	8	Geographical coverage	(i) National: The entire national territory is covered by the surveillance system. (ii) Subnational: Only one or some regions/districts participate in the surveillance system.	If the geographical level is subnational it is important to know the proportion of the population covered.
**Population and cases**	9	Population under surveillance	(i) General population: The population under surveillance is the general population. (ii) Specific population group: The population under surveillance consists of targeted groups based on risk (e.g. injecting drug users for HIV surveillance), surveillance objectives (e.g. hospitalised individuals for surveillance of hospital acquired infections) or other considerations (e.g. surveillance in vulnerable populations such as migrants).	None
	10	Case detection policy	(i) No specific policy: There is no special guidance or policy to identify cases other than diagnoses reported by healthcare providers. (ii) Active case finding: Policy in place for identification and diagnosis of people who may have been exposed to risk factors, e.g. contact tracing. (iii) Screening: Policy in place for systematic examination of asymptomatic people to identify cases, e.g. screening for TB in people living with HIV. (iv) Active case finding and screening: Both policies are in place.	None
	11	Case definition used	Description or reference to the case definition used.	None
	12	Case classification	(i) Confirmed: Only cases who meet the case definition criteria for a confirmed case are reported. (ii) Confirmed-Probable: Cases who meet the case definition criteria for both a confirmed and a probable case are reported. (iii) Confirmed-Probable-Possible: Cases who meet the case definition criteria for a confirmed, a probable and a possible case are reported. (iv) Other: Combination of case classification categories other than the above.	None
	13	Estimate of under-reporting of cases	(i) No estimate of under-reporting: There is no estimate of under-reporting available. (ii) Estimate of under-reporting: There is/are studies estimating the extent of under-reporting.	If there are estimates of under-reporting, collecting information on the studies, i.e. methods, results and interpretation, is recommended.
**Supplementary data**	14	Follow-up data reported	(i) No: Data are reported around the time of recognition/diagnosis of the case; no information is collected on follow-up encounters with health services and/or treatment outcome. (ii) Yes: Data are reported around the time of recognition/diagnosis of the case, and information is reported on follow-up encounters with health services and/or treatment outcome, e.g. TB, HIV in some countries.	None
	15	Outbreak data reported	(i) No: If the case is part of an outbreak, information linking the case with other cases of the same outbreak is not reported. (ii) Yes: If the case is part of an outbreak, information linking the case with other cases of the same outbreak is included in the reporting notification.	None
	16	Molecular typing data reported	(i) No: Laboratory findings reported do not include molecular typing data. (ii) Yes: Laboratory findings reported include molecular typing data.	None
**Information flow**	17	Reporting levels	List of all reporting levels with their respective geographical level, e.g. local, regional, national.	None
	18	Means of healthcare providers reporting to PH authority	(i) Paper-based reporting: Most reports involve filling in printed paper forms that can be sent by any means. (ii) Electronic (non-web) reporting: Most reports involve filling in non-web-based electronic forms that are then sent by email. (iii) Web-based reporting: Most reports involve filling-in web-based electronic forms. (iv) Automated data transfer: Most reports involve automated data transfer from electronic health records and/or laboratory information management systems.	Targets can be set for monitoring progression, e.g. percentage of cases reported electronically.
	19	Frequency of reporting	(i) At time of recognition/diagnosis (ii) Weekly (iii) Monthly (iv) Annually (v) Other	If there are several levels involved with different frequency of reporting, it is recommended to distinguish reporting to local PH authorities from reporting to higher levels, i.e. national or international PH authorities.
**Period of time**	20	Reporting period	Start and end dates for data collection in the surveillance system.	It is recommended to distinguish start and end dates of data collection from reporting if relevant.

PH: public health; TB: tuberculosis.

### Surveillance scheme

This category comprises descriptors of the basic characteristics of the surveillance system, most of which have been categorised somewhat similarly by others. For example, Rothman et al. labelled a similar category ‘Approaches to surveillance’ [1] whereas the WHO put these descriptors under ‘surveillance design characteristics’ [8]. This reflects the difficulty in grouping together strict design elements (e.g. sentinel scheme), description of data sources (e.g. laboratory-based), data format (case-based vs aggregated data) and legal considerations (notifiable diseases vs voluntary reporting). For the system design, we preferred to distinguish comprehensive from sentinel rather than population-based from sentinel as in other descriptions [8]. Population-based refers to systems or studies using a defined population, which allows estimating disease incidence rates [11]. Population-based systems can depend on surveys targeting a representative sample of people or facilities [1]. In addition, it may also be possible to calculate rates with a sentinel scheme. Therefore, we deemed it more consistent to distinguish the inclusion of all facilities from a subset of them than to distinguish a scheme based on population (i.e. population-based) from one based on healthcare providers (i.e. sentinel scheme).

Although the terms of active and passive surveillance are useful conceptually, they may be insufficient for accurately describing the surveillance method in use [1]. We decided to keep them under mode of reporting, but added a type for capturing automated data transfer as the active and passive distinction is no longer relevant when data for all diagnosed cases are automatically transferred from the source, e.g. laboratories, to the surveillance system.

If a primary source collects aggregated data, this format will remain throughout the information flow, e.g. general practitioners reporting weekly aggregated numbers of influenza-like illness cases. However, if case-based data are reported to the local level, these data can be aggregated when reported to a higher level. For example, the surveillance of Zika virus infection in the European Union (EU)/European Economic Area (EEA) allowed reporting of aggregated data to potentially reduce the reporting burden in countries experiencing large local outbreaks [12]. Since the legal status of reporting can be a useful tool to support infectious disease surveillance, we proposed to keep a descriptor for this aspect [13].

Theoretically, surveillance is carried out continuously but in some instances, cases are not reported year-round. The latter is the case for seasonal influenza surveillance in the EU/EEA for which weekly data are reported to national public health authorities from week 40 to week 20 of the following year [14]. This is why we proposed a descriptor on the temporal continuity of the system. Furthermore, the types of ‘ad hoc surveillance system’ and ‘survey’ were added. The former applies to situations in which surveillance is organised on a provisional basis because of a specific event that is likely to last for only a certain period of time, e.g. in temporary refugee camps, after a physical disaster or outbreak [15]. Meanwhile, the latter is relevant when surveillance is carried out through repeated surveys, e.g. point prevalence surveys of healthcare-associated infections and antimicrobial use performed periodically in EU countries [16,17].

The data source(s) of a surveillance system is another important structural characteristic. We decided to distinguish mainly between those where all healthcare providers are required to report, and laboratory surveillance systems, i.e. those based on reporting from laboratories only. We added a further type, ‘specific setting’, for special situations in which only specific types of health services are involved, e.g. primary care services for sentinel influenza-like infection surveillance.

The descriptor ‘type of information reported’ is related to ‘data sources’, but is not identical to it. We decided to include it as a separate descriptor in order to describe explicitly the type of information, clinical, epidemiological or laboratory, required by the surveillance system.

Geographical coverage can be considered national or subnational, i.e. limited to areas where cases are expected to occur. For example, Italy implemented an enhanced surveillance of West Nile virus infection in provinces with evidence of recent animal and vector or human infections [18].

### Population and cases

It is important to distinguish between the data source and the population under surveillance. The population under surveillance is the group of people targeted by the surveillance system [1]. If the data source is hospitals, the population under surveillance can still be the general population. However, this would mean that for diseases with mainly mild presentations, the system would only capture the severe end of the disease and most cases would be missed. For example, dengue surveillance at a tertiary paediatric hospital in Bangkok, Thailand, reported very high proportions of dengue haemorrhagic fever (DHF) (> 50%), which is likely to be an overestimation of the true proportion of DHF [19]. Conversely, if the population under surveillance is any person admitted to hospital, such as in the surveillance of healthcare-associated infections, the data source can include all healthcare providers to capture cases diagnosed after discharge.

Whenever possible, we suggest to design systems that cover a defined population in order to be able to calculate rates. If this population is representative of the national population, information on the proportion of the population covered will allow calculation of notification rates per 100,000 population.

The case detection policy is likely to have an important impact on surveillance data. In 2017, the United Kingdom (UK) accounted for more than half of all chlamydia cases reported in EU/EEA countries. This was probably because of a screening programme targeting 15–24-year-olds in England, which had been in place for 10 years [20]. We suggest to distinguish such systematic screening from active case-finding in which there is a specific strategy to seek persons who were exposed to certain risk factors, for example during an outbreak associated to a food item. Contact tracing is a particular form of case finding for some contagious diseases in which diligent efforts are made to find persons who have been in contact with a known case. For example, most of the EU/EEA countries have a contact tracing strategy for tuberculosis [21]. The intensity with which case finding is carried out can vary, from, for example, one visit or repeated visits to households [22].

On top of the case definition used, it is essential to specify if all cases should be reported to the surveillance system, which we propose to describe under case classification. It may be that criteria defining a possible case are not specific enough for the purpose of surveillance but good enough for an outbreak investigation. Yet, such information is crucial when analysing surveillance data.

Quantification of under-reporting cannot be derived directly from surveillance data. However, this performance indicator, which is sometimes available through specific complementary studies [23], would be valuable when interpreting the data. Therefore, we included it in the proposed set of descriptors. We did not include other performance indicators in the proposed list of surveillance descriptors because these indicators can either be derived from existing surveillance data, e.g. timeliness of case reporting or data completeness for variables included, or require a formal evaluation of the surveillance system, e.g. simplicity, flexibility, positive predictive value etc. [7].

### Supplementary data

For the surveillance of chronic infectious diseases such as HIV/AIDS or tuberculosis, which often involves the collection of longitudinal or follow-up information such as status or treatment outcome, we suggest to specify whether this information is available. For outbreak prone diseases, we propose mentioning information on cluster status, e.g. cases belonging to a known outbreak, which may help interpret unexpected notification peaks. For example, such information helped identify Legionnaires’ disease cases associated with the large outbreak that occurred in Vila Franca de Xira, Portugal, in 2014 [24].

Because of their specific format and their increasing use, we decided to add an indicator on molecular typing data. Whole genome sequencing (WGS) data are expected to become an integral part of infectious disease surveillance systems, and its routine application in food- and waterborne diseases surveillance illustrates this point [25]. However, as the use of WGS data is associated with many challenges including storage, management, analysis and interpretation, additional indicators may be necessary in the future to describe the integration of such data within the various surveillance systems.

### Information flow

In this category, we grouped descriptors that portray basic features of the reporting process to public health authorities in a country. This information can be important for the interpretation of surveillance data, as differences between countries regarding these features (number of reporting levels, means and frequency of reporting) may be a source of differences in notification rates or timeliness of reporting. We decided to make these explicit because the various levels involved in reporting may have different roles in terms of response or analysis. For example, the local public health authority may be in charge of contact tracing or outbreak investigation whereas the national public health authority may prepare annual reports looking at long-term trends and risk factors.

### Period of time

We considered that for the better use of surveillance data, it is important to explicitly state the time period covered by the surveillance scheme. This is because surveillance data are collected for action, but they may also serve other purposes, including research studies. In addition, two surveillance schemes may coexist with different starting dates.

## Conclusion

Surveillance systems were initially designed to detect and help control outbreaks. The concept has evolved and they are now being used for wider purposes, including long-term trends monitoring and informing cost-effectiveness analysis of preventive measures [26]. Such analyses require high quality data and a good understanding of the data sources and their limitations.

To determine whether surveillance systems can meet their objectives, being able to capture and describe their fundamental structural elements is of the utmost importance. Surveillance descriptors should cover all aspects of surveillance systems. The definitions of these descriptors should be as clear and simple as possible to avoid any confusion or misinterpretation of the terms used. The relative importance of each element may vary depending on the objective of the surveillance scheme, but also on the geographical setting (international, national or subnational) and available resources.

Surveillance descriptors should be reviewed periodically to document changes, e.g. increase of geographical coverage, and to assess if a system continues to be fit for purpose.

Together with the minimum requirements for variables, e.g. minimum completeness, and the planned outputs for disseminating the data, surveillance descriptors can be used to define surveillance standards. Such standards may exist for some diseases and settings but they are seldom explicit. We recommend that all surveillance systems have defined surveillance standards based on common and clear surveillance descriptors, for which we present a comprehensive set. We think that such approach would improve both efficiency and acceptability of surveillance.
